# The Effects of Encapsulation on the In Vitro Anti-Clostridial Activity of Olive Mill Wastewater Polyphenolic Extracts: A Promising Strategy to Limit Microbial Growth in Food Systems

**DOI:** 10.3390/molecules29071441

**Published:** 2024-03-23

**Authors:** Rossana Roila, Sara Primavilla, David Ranucci, Roberta Galarini, Fabiola Paoletti, Caterina Altissimi, Andrea Valiani, Raffaella Branciari

**Affiliations:** 1 Department of Veterinary Medicine, University of Perugia, Via San Costanzo 4, 06126 Perugia, Italy; rossana.roila@unipg.it (R.R.); david.ranucci@unipg.it (D.R.); caterina.altissimi@studenti.unipg.it (C.A.); 2 Istituto Zooprofilattico Sperimentale dell’Umbria e delle Marche “Togo Rosati”, Via Salvemini 1, 06126 Perugia, Italy; r.galarini@izsum.it (R.G.); f.paoletti@izsum.it (F.P.); a.valiani@izsum.it (A.V.)

**Keywords:** *Clostridium botulinum*, *Clostridium perfringens*, *Clostridioides difficile*, MIC, MBC, time-kill test, kinetic parameters, circular economy, antioxidant activity, olive oil wastewater extract

## Abstract

Despite the technologies applied to food production, microbial contamination and chemical deterioration are still matters of great concern. In order to limit these phenomena, new natural approaches should be applied. In this context, the present study aimed to assess the antioxidant and anti-Clostridial effects of two different polyphenolic extracts derived from olive mill vegetation water, one liquid (LE) and one encapsulated (EE). The extracts have been preliminary characterized using Liquid Chromatography Quadrupole Time-Of Flight spectrometry. The Oxygen Radical Absorbance Capacity method was used to determine the antioxidant capacity, registering a higher value for EE compared to that for LE (3256 ± 85 and 2446 ± 13 µgTE/g, respectively). The antibacterial activity against *C. perfringens*, *C. botulinum* and *C. difficile* was studied by the agar well diffusion method, MIC and MBC determination and a time-kill test. The results confirm that EE and LE are able to limit microbial growth, albeit with minor effects when the phenolic compounds are encapsulated. Further studies are needed to evaluate the possible application of these extracts in food systems.

## 1. Introduction

Food microbiological contamination is still a matter of great concern worldwide, being closely related to issues like food poisoning and consumers’ health, together with food waste, sustainability and food business cost-effectiveness [1]. Microbial pathogens are the cause of many foodborne diseases, representing a significant safety concern for public health and a pervasive food safety problem [2]. 

*Clostridium botulinum* and *Clostridium perfringens* are the two Clostridia most commonly associated with food borne illnesses [3]. In particular, *C. perfringens* can frequently cause food borne illnesses, and in 2021, *C. perfringens* toxins caused the highest number of cases and deaths among foodborne outbreaks involving toxigenic bacteria [4]. *C. botulinum* is a severe hazard in foodstuffs due to its ability to produce the most poisonous substances known, the botulinum neurotoxin, which is the causative agent of botulism [3]. On the other hand, *Clostridioides difficile* has changed, and some reviews describe an increase in community-associated infection not linked to the traditional risk factors (recent antibiotic therapy, older age, significant comorbidity or previous hospitalization) [5]. In consideration of this aspect, new possible routes of transmission of *C. difficile* have been considered, including the ingestion of contaminated food [5,6].

To counteract the undesirable effect of microbial growth, various food preservation approaches have been developed, namely thermal and chemical methods [7]. The thermal processing of foods can have, in certain conditions, depletive organoleptic and nutritional effects and may not be sufficient against spore-forming bacteria [8]. Synthetic food preservatives instead, like nitrates, benzoates, sulfites, sorbates and formaldehyde, are known for their health-threatening side effects, raising considerable concern among consumers, especially related to long-term exposure [9,10]. In this context, novel sustainable methodologies are required as an alternative to chemical and thermal preservations to inhibit the growth of pathogenic bacteria and prolong the shelf-life of food products, ensuring, therefore, food safety, while not affecting the food quality standards, and representing, at the same time, a sustainable approach [11,12]. These imperatives have led to ever-increasing scientific interest in defining novel biopreservation methods through the possible utilization of some plant extracts as effective natural food preservatives [13,14].

Several studies have shown that agro-industrial by-products are good sources of bioactive compounds with strong antimicrobial effects against food-related microorganisms [13,15,16]. Among the by-products currently under investigation, promising results have been shown by liquid wastes from olive oil production [17]. Olive mill wastewaters (OMWWs) are characterized by a high content of phenolic compounds with known antioxidant and antibacterial activities, such as hydroxytyrosol and tyrosol [17], although little information has been given regarding their efficacy towards specific food-borne microorganisms, especially those belonging to the *Clostridia* genus.

After appropriate purification and extraction, these by-products could represent a valuable source of these mentioned molecules with a great potential for application in food production as natural additives to increase the food quality and safety avoiding the use of chemical preservatives, but also, in some cases, as bioactive ingredients to protect consumers’ health [18]. However, these molecules are likely subjected to degradation processes promoted by environmental and processing conditions oxygen, water, light or other conditions, which limit their efficiency and reduce shelf life [19] For this reason, microencapsulation has been considered a powerful technology for the stabilization of such compounds overcoming the aforementioned issues and enabling their use in the food industry [20]. 

Freeze-drying encapsulation in amorphous carbohydrate microstructure matrices, such as maltodextrin, has been proved to be the most suitable method for drying delicate and thermosensitive biomaterials, minimizing the thermal degradation reactions, chemical changes such as oxidation, and protecting against undesirable physical phenomena (stickiness and collapse) [20,21]. To this end, microencapsulation can be considered a promising technology for overcoming the susceptibility of these compounds to adverse external effects or harmful food processing conditions in order to improve their miscibility and stability during shelf life [22]. A study in the literature proved the efficacy of encapsulated polyphenols in improving products’ shelf-life [22]. 

This type of process also enables the modification of physical properties, allowing for the transformation of liquid extracts, such as OMWW, into powders for easier handling and inclusion in certain types of food matrices [20,23]; additionally, it can be useful to mask unpleasant feelings during eating, such as a bitter taste and the astringency of polyphenols [24].

The aim of this work was to study the in vitro antioxidant and antimicrobial effects against *C. perfringens*, *C. botulinum* and *C. difficile* of two different polyphenolic extracts derived from OMWW, one of which was encapsulated, and the other was pure liquid. The assessment of specific antibacterial activity, as well as the evaluation of the encapsulation impact, will allow for the definition of threshold doses and most suitable formulation for the future application of food models. 

The acquired results could provide the interesting possibility of the reuse of OMWW to be considered not just as a waste material, but instead as an important source of bioactive compounds with antioxidant and antibacterial effects, with a multitude of possible applications addressing the complex issue of food preservation.

## 2. Results

### 2.1. Determination of the Phenolic Profile

**Figure 1:**
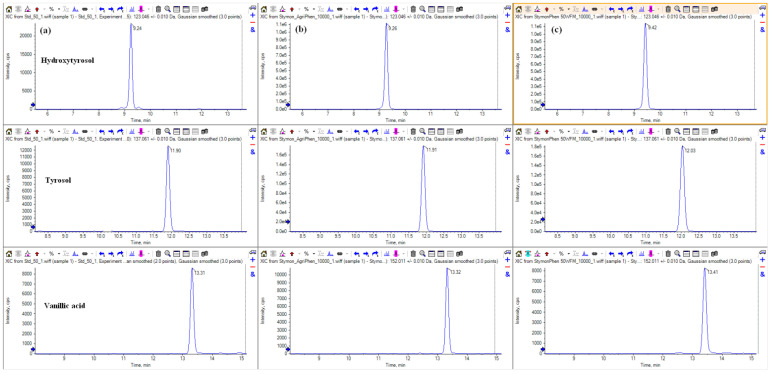
Extract ion chromatograms of the three most abundant polyphenols: (**a**) standard solution at 50 ng/mL; (**b**) liquid extract (10,000-fold dilution); (**c**) encapsulated extract (10,000-fold dilution).

The specific content of major phenolic compounds in LE and EE, as well as their specific chromatograms, are reported in Table 1 and Figure 1.

### 2.2. Antioxidant Activity

The ORAC_FL_ results show a higher antioxidant activity level for EE compared to LE, as the first registered as 3256 ± 85 µgTE/g, and the second was 2446 ± 13 µgTE/g. The encapsulated extract has a higher antioxidant activity level than the liquid extract, most likely due to its higher phenol content.

### 2.3. In Vitro Evaluation of Antibacterial Activity—Agar Well Diffusion and Broth Microdilution 

The in vitro antibacterial activity of EE and LE against the three selected Clostridia was preliminary assessed using an agar well diffusion method, defining the presence and diameter of inhibition zones (Table 2). 

The results reported in Table 2 show that both the encapsulated and liquid polyphenolic extracts exert antibacterial activity against the *Clostridia* strains. In particular, the data suggest that LE has a higher antibacterial activity level at the tested concentrations, as its highest concentration tested (pure) is more powerful than the highest EE concentration tested (1 g/mL) for all the microorganisms (*p* < 0.05). For instance, EE 1 g/mL is comparable to LE 0.558 g/mL for *C. difficile*, slower than LE 0.558 g/mL for *C. botulinum*, and between the value for LE pure and 0.558 g/mL for *C. perfringens.*

Overall, the data reported in Table 2 suggests that the three *Clostridia* species have a slightly different sensitivity to the two extracts, with *C. perfringens* being the most sensitive, followed, in order, by *C. difficile* and *C. botulinum*.

Concerning the results for the broth microdilution method, EE obtained the results reported in Table 3, confirming what was previously reported for the agar well diffusion method in terms of species sensitivity. For these extracts, indeed, the three *Clostridia* tested show a decreasing susceptibility, in the order *C. perfringens*, followed by *C. difficile* and *C. botulinum*. Concerning LE, the assessment revealed the same values of MIC for all the strains, corresponding to 0.070 g/mL. Albeit, these two dilution scales are not directly comparable; 0.070 g/mL represents a smaller amount of extract with respect to 0.125 g/mL. Therefore, it can be inferred that LE has a larger antimicrobial effect on the targeted bacteria tested with the broth microdilution method. It is important to note that in all the cases, the MIC and MBC values are equivalent, suggesting that these extracts are characterized by a bactericidal nature towards Clostridia species.

### 2.4. Time-Kill Test and Evaluation of Growth Dynamics

The microbial growth values (Log CFU/mL) registered at each point of the time-kill test for the targeted Clostridia under the effect of EE and LE compared to those of the CTRL are reported in Table 4. The results confirm that both the olive mill wastewater extracts (EE and LE) are able to decrease the microbial proliferation of all the bacteria starting as early as the fourth hour of testing (*p* < 0.05). Particularly, the liquid extract (LE) seems to possess a higher antibacterial activity level as, for all the three strains, the limit of quantification (LOQ = −0.50 Log CFU/mL) is reached at 8 h, while for the encapsulated extracts (EE), this is reached slightly later at 12 h. 

Overall, from the beginning of the test (time 0) to the end (time 48), on average, the growth control (CTRL) has a ∆ Log CFU/g of 2.49, while for LE and EE, these are −5.85 and −5.84, respectively.

**Figure 2:**
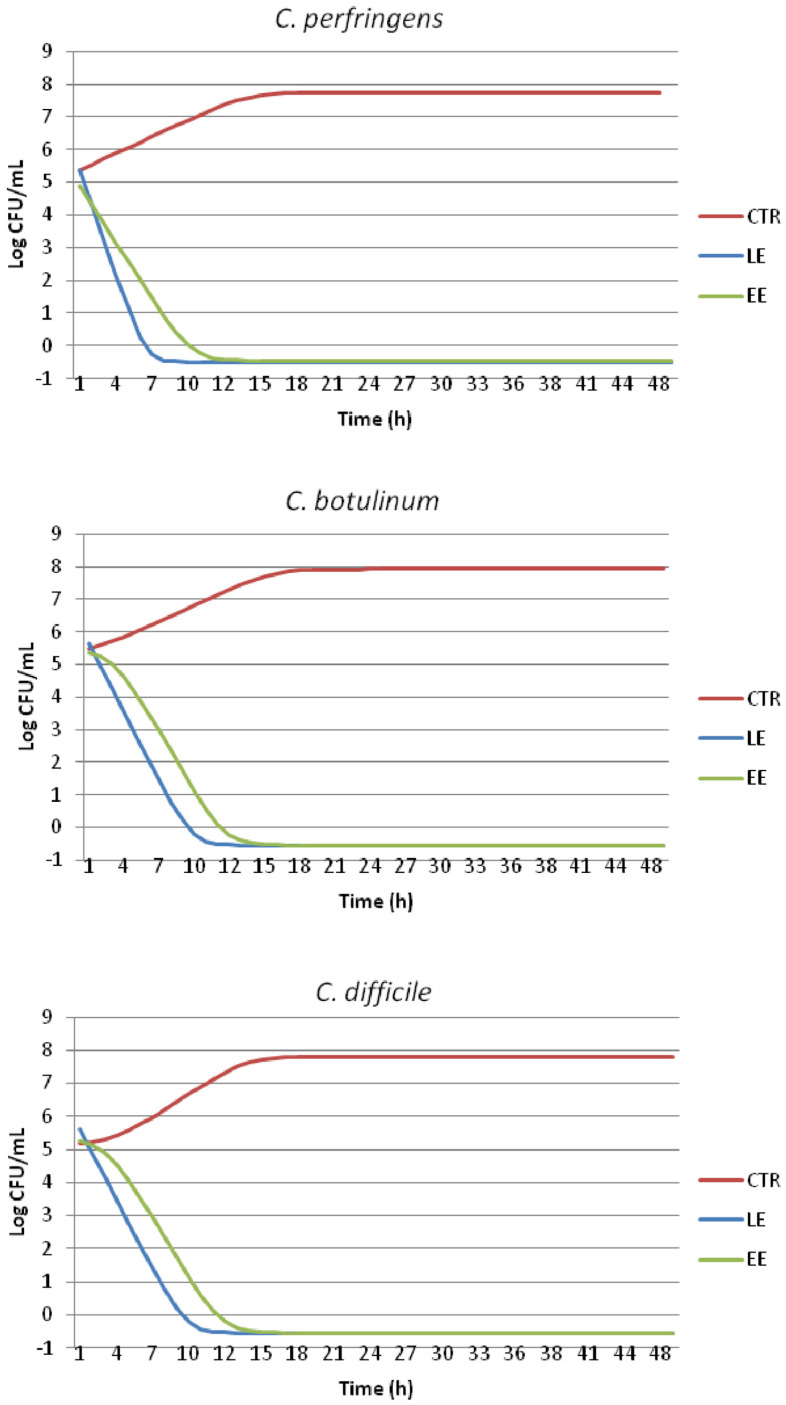
Modeled growth curves of *C. perfringens*, *C. botulinum* and *C. difficile* cultured with encapsulated polyphenolic extract (EE) and the liquid polyphenolic extract (LE) compared to that of CTRL samples.

This outcome is also shown in Figure 2, representing the estimated growth curves of the tested bacteria cultured with the addition of EE and LE compared to that of the CTRL. The curves constructed using ComBase DMfit represent the best fitting of the raw data reported in Table 4, allowing for a modeled quantification of microbial responses through the definition of the growth kinetic parameters (Table 5).

Both Figure 2 and Table 5 confirm what was previously highlighted by the analytical data (Table 4) as for all the three bacteria tested, the growth curves of the polyphenolic liquid extract (LE) show no lag phase and drop quickly after T0, reaching the LOQ earlier compared to EE. Furthermore, the maximum growth rate is always lower for LE compared to that of EE.

The fitted curves of the CTRL show the rapid proliferation of microorganisms to a final value of almost 8 Log CFU/mL at around 20 h of testing, followed by a stationary phase.

## 3. Discussion

Consumers’ preference has recently changed towards minimally processed foodstuffs characterized by a low salt concentration, the absence of additives, a bland heat treatment and vacuum packaging [25]. As a consequence, a possible drawback is represented by an increased risk of food-borne diseases onset (e.g., botulism) and the quicker deterioration of food characteristics (e.g., lipid and protein oxidation), resulting in a shorter shelf-life [25,26]. In order to counteract this phenomenon, novel antimicrobial and antioxidant control measures should be applied [26]. 

Recently, great interest has been paid to natural antimicrobial and antioxidants in the food industry as they can be used as technological strategies to improve the quality of foodstuffs [26,27]. These molecules can potentially be applied directly to food products or by coating packaging materials to improve products’ oxidative and microbial stability, thus avoiding or reducing the use of chemical compounds [26,27]. This approach could also mitigate consumers’ perceptions of the risks related to the food chain, meeting their increasing preference towards more healthy and clean-labeled food products [9,10].

The antioxidant activity registered for the tested olive mill waste water extracts with the ORAC_FL_ method is in agreement with the previous results on similar extracts [26], confirming the powerful antioxidant activity of olive-derived phenolic compounds [26,28]. It has been observed that phenols act as hydrogen donors and that the o-dihydroxyl group possesses a high antioxidant activity level, counteracting the free radicals [29].

In the literature, the antioxidant properties of olive mill waste water extracts have also been tested when directly added into foodstuffs. A previous study shows that these types of polyphenolic extracts exert a higher antioxidant activity level, even when compared with a commercial antioxidant additive, such as ascorbic acid [26]. Particularly, the greater effect was observed for lipid oxidation, thus improving the product’s shelf life [26]. Oxidation has been demonstrated as the main non-microbial cause of food quality deterioration [30]. For instance, oxidative deterioration is capable to limit food acceptability and shortening its shelf-life by causing discoloration, the development of off-flavors and the formation of toxic compounds [28].

It has been hypothesized that this high antioxidant activity level of the olive mill waste water extracts is mainly attributable to hydroxytyrosol due to its chemical structure, which includes a phenol ring formed by a catechol group and three hydroxyl groups [29]. For instance, Martínez-Zamora et al. [31] observed the reduction in meat products’ oxidation when incorporating synthetic hydroxytyrosol to the recipe. However, the highest activity level is reached in synergy with other molecules contained in natural extracts [17,26], such as EE and LE.

Olive polyphenols, such as hydroxytyrosol, have been shown to have high levels of antioxidant activity, as have some plant extracts [32] and fruit by-products [33], making them useful in a variety of market segments, including the pharmaceutical sector and, in particular, the food industry, where they can be used to produce functional foods or as natural additives to prevent lipid oxidation and microbial alteration [34].

The in vitro anti-Clostridial effect of different phenolic compounds has already been reported in the literature. A previous study by Primavilla et al. [35] refers to the similar antibacterial activity of *Crocus sativus* L. extract (1 g/mL) assessed by agar well diffusion, albeit *C. botulinum* and *C. perfringens* seemed more sensitive than *C. difficile*. This diverse outcome is of no surprise as the different chemical composition and formulation of the extracts can lead to different results of microbial growth modulation.

Concerning the effects of similar natural compounds, Dağdelen [36] investigated the antimicrobial activities of the phenolic extracts of virgin olive oils and found that those extracts rich in hydroxytyrosol, tyrosol and vanillic acid were able to exert strong antibacterial activity against Clostridia, specifically *C. perfringens.*

Medina [37], who largely studied the antibacterial activity of olive-derived polyphenols, reported strong bactericidal activity against *C. perfringens* exerted by hydroxytyrosol and tyrosol, and both compounds are present in the two tested extracts.

The antimicrobial activity of vanillic acid has previously been reported elsewhere [38,39]; however, the data lack information on its efficacy against *Clostridioides*.

As mentioned above, the results concerning the broth microdilution method show the same concentration of OMWW-derived extracts for MIC and MBC in all the cases considered, suggesting, as reported elsewhere [40], that these extracts are characterized by a bactericidal nature towards some microorganisms, including *Clostridia* species. The MIC/MBC obtained in the present study are in agreement with some other studies testing the antimicrobial activity of olive-derived polyphenolic extracts, showing similar values, albeit for other bacteria [41]. In the literature, the anti-Clostridial activity of bioactive extracts of turmeric was assessed against *C. difficile* that was inhibited at concentrations ranging from 4 to 32 μg/mL [42], which are lower than those reported in the present study.

Concerning the time-kill test, in a previous study testing saffron natural extracts on the same bacterial strains, the LOQ was reached at around 24 h, reflecting different antibacterial actions most certainly due to the different chemical compositions of the extracts [35]. Manuka (*Leptospermum scoparium*) honey was reported as having in vitro bactericidal activity and a therapeutic role in the prevention of *C. difficile* infection [43], in addition to potent biofilm inhibition activity in vitro [44].

Albeit the mechanism of action of OMWW compounds has not been completely elucidated, a recent study aiming to investigate some natural products with bactericidal effects, such as cinnamon root powder and peppermint oil against *C. difficile* strains, revealed that cytoplasmic membrane damage was recognized as the mechanism of action mainly responsible for the activity of several natural products against bacteria, in addition to ATP (adenosine triphosphate) and protein leakage [45,46]. This finding is in line with the previous studies hypothesizing this mechanism of action of OMWW for other microorganisms [47].

The results of the present study indicate that this encapsulated extract has a slightly smaller antimicrobial effect against the Clostridia tested compared to that of the liquid one. This finding is in agreement with other studies reporting that pure extracts exert superior antimicrobial activity due to the direct release of active chemical compounds [48]. Contrarily, in some other experiments, the antibacterial activity level of natural compounds was increased after encapsulation [49]. 

In the literature, polyphenolic extracts were shown to have anti-Clostridial activities, preventing vegetative cell growth or spore germination [25]. This aspect has not been investigated in the present study, but the specific effect of polyphenolic compounds on spore survival and germination could be an interesting topic for future research. 

The effects of encapsulation on extracts’ antimicrobial capacity, such as the decrease, increase or conservation of their bioactivity, depend on different aspects, including the interactions established between the functional groups of the encapsulated compound and the encapsulating material, as well as interaction with the matrix [1]. Therefore, albeit different polyphenolic extracts have already been experimentally added to foodstuffs [50,51], it is crucial to assess the applicability of food-grade OMWW polyphenolic extracts, both encapsulated and pure, in specific complex food systems, aiming to provide them with improved characteristics. Consumers’ attitudes, perceptions and behaviors towards these products have changed in recent years, and their preferences are shifting towards “natural” compounds for a healthier lifestyle. These attitudes emphasize the responsibility of industries and national authorities to provide a safer product, albeit, currently, there are no specific regulations or legal guidelines on the use of such products. Nevertheless, bioactive compounds must be assessed for any hazard to human health and meet legal requirements by force before entering the market [52].

It is also worth noting that recovering olive phenols from wastewater can make a significant contribution to environmental protection, as their dispersion in soil poses an environmental threat, while the water after phenolic extraction is suitable for irrigation. For this reason, the purification and extraction of biomolecules make it very appealing to use OMWW as a starting material for a large variety of applications, including food production [27].

## 4. Materials and Methods

### 4.1. Test Materials

OMWW extracts enriched in polyphenols were provided by Stymon Natural Products P.C., Patras, Greece (www.stymon.com, accessed on 20 November 2023). This product derives from OMWW (*Olea europaea* L., Koroneiki cultivar). The extracts tested were formulated in two different products, liquid (not encapsulated) and powder encapsulated extract (EE). EE was treated with hydrolytic enzymes, filtered by a membrane process, and encapsulated with a food-grade maltodextrin carrier (1:1 dw), followed by lyophilization (freeze-drying technique at −55 °C, 0.1 mbar) and a grinding process. The above two products were produced via a patented process (GR1010150, EP4049543A1) using green technologies. The extract was chosen by the authors because it is food-grade (certificate released by General Chemical State Laboratory of Greece n. 30/003/000/3810), and the treatment was carried out using only water without the addition of any chemicals. 

The concentrations of both polyphenol extracts were measured by Liquid Chromatography Quadrupole Time-Of Flight spectrometry (LC-QTOF-LC-TripleTOF 6600, Sciex, Framingham, MA, USA). Acetonitrile (ACN) and methanol were LC-MS-grade and supplied by Carlo Erba Reagents (Milan, Italy). Dimethyl sulfoxide (DMSO) was provided by VWR International (Radnor, PA, USA). Butylated hydroxytoluene (BHT) was purchased from Alfa Aesar (Haverhill, MA, USA), and acetic acid (99%) was bought from Merck (Darmstadt, Germany). Deionized water was produced using a Milli-Q purification system (Millipore, Molsheim, France). Hydroxytyrosol and verbascoside were purchased from Extrasynthese (Genay, France). Oleuropein aglycone was supplied by TRC (Toronto, ON, Canada). Tyrosol, vanillic acid, vanillin, *p*-coumaric acid, oleuropein, pinoresinol, luteolin and apigenin were obtained from Merck. Individual standard stock solutions (1 mg mL^−1^) were prepared in methanol, except apigenin, which was prepared in methanol/DMSO 70/30 (*v*/*v*). Stock solutions were stored in the freezer (−20 °C) for six months. By mixing the appropriate amount of individual stock solutions, a working standard mixture at 10 µg mL^−1^ was obtained in methanol. From the latter, the calibration curve (25, 50, 100, 250 and 500 ng mL^−1^) was measured by diluting it with a mixture of acetic acid 0.025%/methanol (90/10, *v*/*v*).

The encapsulated polyphenolic extract (EE) was analyzed starting from 1 g (1.0 g ± 0.1 g) of sample, which was mixed with 5 mL of a methanol/water 80/20 (*v*/*v*) solution containing 20 mg/L of BHT. The extraction was repeated a second time, and after centrifugation, three aliquots of the extract were diluted 1000-, 2500- and 5000-fold, respectively, with a mixture of acetic acid 0.025%/methanol 90/10 (*v*/*v*). Then, each diluted aliquot was injected into the instrumental platform. 

For liquid extract (LE) analysis, 0.1 g (1.0 g ± 0.1 g) of the sample was solubilized with 10 mL of methanol/water 80/20 (*v*/*v*) solution containing 20 mg/L of BHT. Three aliquots were then diluted 1000-, 2500- and 5000-fold, respectively, with a mixture of acetic acid 0.025%/methanol 90/10 (*v*/*v*) and injected.

Instrumental analysis was performed by Liquid Chromatography Quadrupole Time-Of Flight spectrometry (LC-QTOF), determining eleven polyphenols. The platform consisted of an Exion LC™ coupled to a 6600+ TripleTOF™ (AB Sciex, Foster, CA, USA) equipped with an electrospray ionization source operating in negative mode (ESI-). Chromatographic separation was carried out using an Acquity BEH C18 (150 mm × 2.1 mm, 1.7 µm, Waters, Milford, MA, USA). Water with 0.025% acetic acid (A) and methanol/ACN 90/10 *v*/*v* % (B) were the mobile phases. The gradient started with 0% of B (1 min); then, the percentage of B was increased to 20% after 10 min, followed by an increase to 50% B after 4 min and another one to 100% after 3 min. After 5 min, the percentage of B was restored to the initial conditions (0%) in 1 min. Finally, the system was re-equilibrated for 6 min (run time: 30 min). The column temperature was set at 40 °C, and the autosampler temperature was kept at 25 °C. Flow rate and injection volume were 0.25 mL/min and 10 µL, respectively. Compressed air was used as GS1 (55 psi) and GS2 (55 psi), whereas nitrogen was the curtain gas (40 psi). The spray voltage was set at −4.5 kV, and the interface source temperature was set at 450 °C. Single infusions of each analyte were carried out to optimize the declustering potential and collision energy. The precursor ([M − H^+^]^−^) and fragment ions acquired in MRM^HR^ (multiple reaction-monitoring high resolution) mode are listed in Table 6. Mass error was <5 ppm.

### 4.2. Antioxidant Capacity Determination

The antioxidant capacity of polyphenolic extracts was measured using the Oxygen Radical Absorbance Capacity method (ORAC_FL_). This test is based on the decay rate of the fluorescence probe due to radical oxygen species (ROO) compared to the reference standard, Trolox (6-hydroxy-2,5,7,8-tetramethylchroman-2-carboxylic acid, Sigma-Aldrich, Steinheim, Germany). One mL of LE or 1 g of EE was mixed with a buffer, 75 mM, at pH 7.2, containing 13.19 g of K_2_HPO_4_ and 10.26 g of KH_2_PO_4_ in 900 mL of deionized water. The obtained mixture was homogenized for 1 min with an Ultra-Turrax homogenizer (Ultra Turrax T25 Basic, IKA Labortechnik Janke & Kunkel GmbH, Stavfen, Germany), and then vortexed for 2 min. After centrifugation at 6000 rpm for 20 min at +4 °C, the supernatant was used for antioxidant capacity determination. ORAC_FL_ assays were executed on an FLUO-star OPTIMA microplate fluorescence reader (BMGLABTECH, Offenburg, Germany) at excitation and emission wavelengths of 485 and 520 nm, respectively. The results of the test are expressed as µg of Trolox equivalents (TE) per g of sample.

### 4.3. In Vitro Evaluation of Antibacterial Activity—Agar Well Diffusion

The agar well diffusion method was used to screen the antibacterial activities of the extracts against *Clostridium perfringens*, *Clostridium botulinum* and *Clostridioides difficile* [53]. For this test, EE was suspended, and then diluted as needed in demineralized sterile water, while LE was used pure or diluted with demineralized sterile water when necessary.

*C. perfringens* and *C. difficile* strains were derived from the Istituto Zooprofilattico Sperimentale dell’Umbria e delle Marche “Togo Rosati” food isolates collection, while *C. botulinum* was provided by the Italian National Institute of Health (National Referral Center for Botulism). 

As previously described [35], 100 µL of bacteria suspensions (0.5 McFarland in 0.9% sterile saline solution) was spread using a swab on Mueller Hinton agar 5% defibrinated sheep blood plates (MHAB—Oxoid Limited, Basingstoke, UK). Two-fold serial dilutions were prepared for each extract (EE: from 1 g/mL to 0.125 g/mL; LE: from pure to 0.140 g/mL), and 50 µL of each of them was inoculated in 7 mm holes previously made by scooping out the medium with a sterilized cork borer. Negative (sterile demineralized water) and positive controls (Penicillin G 10UI discs—Oxoid Limited, Basingstoke, UK) were set up. Plates were incubated as indicated in Table 7, and after incubation, the diameters of the inhibition halos were measured with a gauge and expressed in mm. The experiments were repeated thrice; mean values and standard deviations of the diameters were calculated.

### 4.4. MIC/MBC Assay

The evaluation of the minimal inhibitory concentration (MIC) and of the minimal bactericidal concentration (MBC) of the extracts was performed in triplicate using a standard broth microdilution technique, as described in the Clinical Laboratory Standards Institute (CLSI) guidelines [54]. Briefly, a decreasing concentration of each extract (ranging from 0.5 g/mL to 0.004 g/mL for EE and from 0.558 g/mL to 0.004 g/mL for LE) was added to an equal volume of bacterial suspensions of 10^5^ colony forming unit/ mL (CFU/mL) in Mueller Hinton broth with 5% blood (Biolife Italiana s.r.l., Milan, Italy) in a 96-well microplate (Starlab International GmbH, Hamburg, Germany). In order to check the experiment, three different control were set up as follows: antibiotic control (bacterial suspension and decreasing concentration of benzylpenicillin sodium salt; Sigma-Aldrich, St. Louis, MO, USA), organism control (bacterial suspension) and negative control (extract solution at the same decreasing concentrations tested). The microplates were incubated for 48 h at 37 °C under anaerobic conditions, and the MIC was determined by evaluating the lowest concentration with no visible bacterial growth [54], while the MBC, was figured out by subculturing 10 μL of wells corresponding to the MIC and to higher MIC concentrations onto 5% Sheep Blood agar dishes (Microbiol s.r.l., Cagliari, Italy). The sheep blood plates were then incubated at 37 °C for 48 h [40]. The MIC and MBC values are expressed as means ± standard deviations. 

### 4.5. Time-Kill Test and Evaluation of Growth Dynamics

As described in the Clinical & Laboratory Standards Institute (CLSI) guidelines (document M26-A) [54], three broth cultures were set up (10^5^ CFU/mL in Mueller Hinton broth with 5% blood; Biolife Italiana s.r.l., Milan, Italy). The first two broth cultures were respectively added to the EE and LE extracts (1 × MIC), while the third one was used as a growth control (CTRL). The broth cultures were incubated at 37 °C under anaerobic conditions using anaerobic jars (2.5 L AnaeroJar, AG002, together with AnaeroGen 2.5 L, AN0025—Oxoid Limited, Basingstoke, UK). At regular time intervals (0, 4, 8, 12, 24 and 48 h), the count of viable cells was performed. The results are expressed as Log CFU/mL, and in case of no bacterial growth, at the 1.00 Log CFU/mL level (<10 CFU/mL), a value of −0.50 Log CFU/mL was assigned [40]. The experiments were repeated thrice, and standard deviations were determined. 

*C. perfringens*, *C. botulinum* and *C. difficile* growth curves and their kinetic parameters (initial value, lag phase duration, maximum growth rate and final value) were defined using the DMFit tool of ComBase’s, free predictive microbiology software (https://www.combase.cc/index.php/en/DMFit—2021 version, accessed on 11 October 2023), by fitting the experimental data to Baranyi and Roberts’ model [55]. 

### 4.6. Statistical Analysis

ANOVA (analysis of variance) was performed with the data obtained from the agar well diffusion and the time kill-test using SAS’s generalized linear model (GLM) approach (SAS Institute Inc., Cary, NC, USA, 2001) [56]. For the first experiment, a mixed model with treatments (EE, LE, and CTRL) and microorganisms as fixed factors were utilized, and for the second test, the same model was employed, with treatments (EE, LE, and CTRL) and time (T0, T4, T8, T12, T24 and T48) as fixed factors. Duplicate effects were determined to be insignificant and were deleted from the model. Tukey’s post hoc analysis was used to explain significant mean differences (*p* < 0.05).

The effects of extracts’ formulation on the growth curve parameters were assessed using the one-way ANOVA model with treatment (EE, LE, and CTRL) as a fixed factor, and Tukey’s test (*p* < 0.05) was used to determine significant differences among the results.

## 5. Conclusions

To ensure microbiological safety and improve the quality of foodstuffs in accordance with consumers’ preferences for “clean label” products, food corporations are looking for new strategies to replace the traditional preservatives. Polyphenolic compounds are among the most well-liked candidates for natural food additives, and the application of encapsulation techniques could address the stability, solubility and bioavailability issues. The results demonstrate a promising antioxidant capacity and anti-Clostridial effect for both the tested polyphenolic extracts derived from olive mill vegetation water, especially for the liquid one. Further investigations are needed to evaluate and refine the possible applications of these extracts in complex food systems in order to provide consumers with safer, healthier and greener products. Additional studies should focus on the evaluation of the conformity and applicability of food-grade encapsulated polyphenolic extracts in the context of real food processes and products, as well as clarify their molecular mechanisms of action.

## 6. Patents

Stymon Natural Products P.C., Patras, Greece (www.stymon.com, accessed on 20 November 2023): patented process (GR1010150, EP4049543A1) and food-grade certificate (certificate released by General Chemical State Laboratory of Greece n. 30/003/000/3810).

## Figures and Tables

**Table 1:** Phenolic contents of the liquid and encapsulated extract (mg/g).

	Phenolic Compounds			Polyphenol Sum
	Hydroxytyrosol	Tyrosol	Vanillic Acid	
EE	14.71 ± 0.27	3.95 ± 0.17	0.26 ± 0.01	18.9
LE	8.58 ± 0.19	2.13 ± 0.05	0.21 ± 0.10	10.9

**Table 2:** Diameters of inhibition halos (mm) obtained when testing the encapsulated polyphenolic extract (EE), the polyphenolic liquid extract (LE) and the Penicillin G-positive control (PC) for the three targeted *Clostridia*. Values are expressed as means of three determination ± standard deviation.

	EE (g/mL)				LE (g/mL)				PC
	1	0.5	0.25	0.125	Pure	0.558	0.279	0.140	
*C. difficile*	10.94 ± 0.10 ^bB^	8.7 ± 0.45 ^aA^	-	-	18.58 ± 0.38 ^bC^	11.38 ± 0.42 ^bB^	7.88 ± 0.12 ^aA^	-	31.44 ± 1.12 ^aD^
*C. botulinum*	8.1 ± 0.20 ^aA^	-	-	-	17.51 ± 0.27 ^aC^	10.66 ± 0.47 ^aB^	-	-	32.24 ± 0.98 ^bD^
*C. perfringens*	17.01 ± 0.37 ^cD^	14.37 ± 0.49 ^bC^	12.19 ± 0.33 ^B^	-	18.99 ± 0.06 ^bE^	13.85 ± 0.29 ^cC^	8.92 ± 0.39 ^bA^	-	32.91 ± 0.70 ^bF^

Different letters in the same row (A,B,C,D,E,F) indicate differences between mean values of halos diameter for the two extracts and the control group (*p* < 0.05); different letters in the same column (a,b,c) indicate differences between mean values of halos diameter for different micro-organisms within the same concentration of extract (*p* < 0.05); positive control = Penicillin G disc.

**Table 3:** Minimum inhibitory concentration (MIC) and minimum bactericidal concentration (MBC) of encapsulated polyphenolic extract (EE), liquid polyphenolic extract (LE) and of the Benzylpenicillin positive control (PC) against *C. perfringens*, *C. botulinum* and *C. difficile*.

	EE (g/mL)		LE (g/mL)		PC (µg/mL)	
	MIC	MBC	MIC	MBC	MIC	MBC
*C. difficile*	0.250	0.250	0.070	0.070	6 × 10^−8^	6 × 10^−8^
*C. botulinum*	0.500	0.500	0.070	0.070	5 × 10^−7^	5 × 10^−7^
*C. perfringens*	0.125	0.125	0.070	0.070	1 × 10^−6^	1 × 10^−6^

**Table 4:** Results of the time-kill test for the targeted microorganisms. Values are expressed as means of three determinations ± standard deviations (Log CFU/mL).

Time (h)	Treatment	*C. perfringens*	*C. botulinum*	*C. difficile*
0	CTRL	5.43 ± 0.03 ^A^	5.52 ± 0.01 ^A^	5.21 ± 0.04 ^A^
	LE	5.36 ± 0.02 ^C^	5.36 ± 0.04 ^C^	5.32 ± 0.04 ^C^
	EE	5.36 ± 0.04 ^D^	5.38 ± 0.02 ^D^	5.28 ± 0.05 ^D^
4	CTRL	5.41 ± 0.05 ^cA^	5.87 ± 0.03 ^cB^	5.46 ± 0.12 ^cB^
	LE	1.00 ± 0.00 ^aB^	3.32 ± 0.00 ^aB^	3.30 ± 0.06 ^aB^
	EE	1.49 ± 0.20 ^bC^	3.95 ± 0.17 ^bC^	3.92 ± 0.06 ^bC^
8	CTRL	6.95 ± 0.01 ^cB^	6.95 ± 0.01 ^cC^	6.76 ± 0.03 ^cC^
	LE	−0.50 ± 0.00 ^aA^	−0.50 ± 0.00 ^aA^	−0.50 ± 0.00 ^aA^
	EE	0.94 ± 0.03 ^bB^	1.60 ± 0.56 ^bB^	1.65 ± 0.16 ^bB^
12	CTRL	7.25 ± 0.05 ^bC^	7.25 ± 0.07 ^bD^	7.27 ± 0.08 ^bD^
	LE	−0.50 ± 0.00 ^aA^	−0.50 ± 0.00 ^aA^	−0.50 ± 0.00 ^aA^
	EE	−0.50 ± 0.00 ^aA^	−0.50 ± 0.00 ^aA^	−0.50 ± 0.00 ^aA^
24	CTRL	7.71 ± 0.02 ^bD^	7.90 ± 0.03 ^bE^	7.79 ± 0.03 ^bE^
	LE	−0.50 ± 0.00 ^aA^	−0.50 ± 0.00 ^aA^	−0.50 ± 0.00 ^aA^
	EE	−0.50 ± 0.00 ^aA^	−0.50 ± 0.00 ^aA^	−0.50 ± 0.00 ^aA^
48	CTRL	7.82 ± 0.03 ^bE^	7.96 ± 0.04 ^bE^	7.83 ± 0.03 ^bE^
	LE	−0.50 ± 0.00 ^aA^	−0.50 ± 0.00 ^aA^	−0.50 ± 0.03 ^aA^
	EE	−0.50 ± 0.00 ^aA^	−0.50 ± 0.00 ^aA^	−0.50 ± 0.03 ^aA^

Considering each of the tested microorganisms, different letters within the sampling time for different treatments (a,b,c) and within each treatment during sampling times (A,B,C,D,E) denote statistical difference (*p* < 0.05). EE = encapsulated polyphenolic extract; LE = liquid polyphenolic extract; CTRL = growth control.

**Table 5:** Dynamic parameters (initial value—Log CFU/mL; lag phase—h; maximum growth rate—Log CFU/mL/h; final value—Log CFU/mL) and regression diagnostics of the modeled growth kinetics of *C. perfringens-*, *C. botulinum-* and *C. difficile*-treated encapsulated polyphenolic extract (EE) and the liquid polyphenolic extract (LE) compared to those of CTRL samples.

	EE	LE	CTRL
*C. perfringens*			
Initial value	4.91 ± 0.07	5.36 ± 0.02	5.27 ± 0.16
Lag phase	-	-	1.76 ± 2.91
Max growth rate	−0.61 ± 0.01 ^b^	−1.102 ± 0.01 ^aA^	0.31 ± 0.22 ^c^
Final value	−0.45 ± 0.03 ^aA^	−0.50 ± 0.01 ^a^	7.71 ± 0.09 ^bA^
R^2^ value	0.891	0.983	0.892
Standard Error of Fit	0.750	0.020	0.357
*C. botulinum*			
Initial value	5.51 ± 0.13	5.64 ± 0.04	5.48 ± 0.03
Lag phase	2.05 ± 0.07 ^b^	-	1.31 ± 0.01 ^a^
Max growth rate	−0.61 ± 0.04 ^b^	−0.74 ± 0.01 ^aB^	0.16 ± 0.014 ^c^
Final value	−0.56 ± 0.01 ^aB^	−0.56 ± 0.00 ^a^	7.93 ± 0.03 ^bC^
R^2^ value	0.989	0.962	0.956
Standard Error of Fit	0.262	0.507	2.215
*C. difficile*			
Initial value	5.26 ± 0.04	5.60 ± 0.03	5.17 ± 0.05
Lag phase	2.13 ± 0.20 ^a^	-	2.45 ± 0.33 ^b^
Max growth rate	−0.64 ± 0.02 ^a^	−0.74 ± 0.01 ^aB^	0.24 ± 0.02 ^b^
Final value	−0.55 ± 0.01 ^aB^	−0.56 ± 0.01 ^a^	7.79 ± 0.03 ^bB^
R^2^ value	0.996	0.960	0.961
Standard Error of Fit	0.161	0.510	0.225

Different letters in the same row (a,b,c) indicate differences between mean values for the two extracts and the control group (*p* < 0.05); different letters in the same column (A,B,C) indicate differences between mean values for different microorganisms within the same treatment (EE, LE or CTRL) (*p* < 0.05).

**Table 6:** Retention times (RTs) and monitored ions acquired using LC-QTOF in multiple reaction-monitoring high-resolution modes.

Analyte	RT (min)	Molecular Formula	Precursor Ion (*m*/*z*)	Fragment Ion (*m*/*z*)	Declustering Potential (V)	Collision Energy (V)
Hydroxytyrosol	9.2	C_8_H_10_O_3_	153.0557	123.0455	−80	−14
Tyrosol	11.9	C_8_H_10_O_2_	137.0608	119.0520	−90	−18
Vanillic acid	13.3	C_8_H_8_O_4_	167.0350	152.0111	−70	−15
Vanillin	14.9	C_8_H_8_O_3_	151.0401	136.0166	−60	−14
*p*-Coumaric acid	15.4	C_9_H_8_O_3_	163.0401	119.0500	−60	−14
Verbascoside	16.4	C_29_H_36_O_15_	623.1981	161.0251	−90	−38
Oleuropein	17.3	C_25_H_32_O_13_	539.1770	307.0824	−100	−27
Pinoresinol	17.4	C_20_H_22_O_6_	357.1344	151.0410	−80	−20
Luteolin	17.5	C_15_H_10_O_6_	285.0405	133.0293	−110	−36
Oleuropein aglycone	17.6	C_19_H_22_O_8_	377.1242	307.0824	−80	−14
Apigenin	17.7	C_15_H_10_O_5_	269.0456	117.0343	−110	−35

**Table 7:** Incubation times and temperatures and positive control used for the agar well diffusion test.

Microorganisms	Growth Conditions	Positive Controls
*Clostridium perfringens*	37 °C—24–48 h under anaerobic conditions * in MHAB	Penicillin G 10UI/disc
*Clostridium botulinum* ISS CNRB CL 14NT	37 °C—24–48 h under anaerobic conditions * in MHAB	Penicillin G 10UI/disc
*Clostridioides difficile*	37 °C—24–48 h under anaerobic conditions * in MHAB	Penicillin G 10UI/disc

* anaerobic jars (2.5 L AnaeroJar, AG0025 with AnaeroGen 2.5 L, AN0025—Oxoid Limited, Basingstoke, UK).

## Data Availability

The data are contained within this article.
